# Dietary preferences and feeding strategies of Colombian highland woolly monkeys

**DOI:** 10.1038/s41598-022-17655-5

**Published:** 2022-08-23

**Authors:** Manuel L. Fonseca, Marcela A. Ramírez-Pinzón, Kaylie N. McNeil, Michelle Guevara, Laura M. Gómez-Gutiérrez, Klaus Harter, Alvaro Mongui, Pablo R. Stevenson

**Affiliations:** 1 Laboratorio de Ecología de Bosques Tropicales y Primatología (LEBTYP), Departamento de Ciencias Biológicas, Universidad de Los Andes, Bogotá, Colombia; 2 Comparative Zoology, Institut für Evolution und Ökologie (EvE), Eberhard Karls Universität Tübingen, Tübingen, Germany; 3 Laboratorio de Zoología y Ecología Acuática (LAZOEA), Departamento de Ciencias Biológicas, Universidad de Los Andes, Bogotá, Colombia; 4 Department of Anthropology, University of Texas at San Antonio, San Antonio, TX USA; 5 Departamento de Ciencias Biológicas, Facultad de Ciencias, Universidad de los Andes, Bogotá, Colombia; 6 Zentrum für Molekularbiologie der Pflanzen (ZMBP), Eberhard Karls Universität Tübingen, Tübingen, Germany

**Keywords:** Molecular biology, Animal behaviour, Entomology, Ecology, Behavioural ecology, Molecular ecology

## Abstract

Primates are very selective in the foods they include in their diets with foraging strategies that respond to spatial and temporal changes in resource availability, distribution and quality. Colombian woolly monkeys (*Lagothrix lagotricha lugens*), one of the largest primate species in the Americas, feed mainly on fruits, but they also eat a high percentage of arthropods. This differs from closely related Atelid species that supplement their diet with leaves. In an 11 month study, we investigated the foraging strategies of this endemic monkey and assessed how resource availability affects dietary selection. Using behavioural, phenological, arthropod sampling and metabarcoding methods, we recorded respectively foraging time, forest productivity, arthropod availability in the forest and arthropod consumption. Scat samples and capturing canopy substrates (i.e. moss, bromeliads, aerial insects) were used for assigning arthropod taxonomy. The most important resource in the diet was fruits (54%), followed by arthropods (28%). Resource availability predicted feeding time for arthropods but not for fruits. Further, there was a positive relationship between feeding time on fruits and arthropods, suggesting that eating both resources during the same periods might work as an optimal strategy to maximize nutrient intake. Woolly monkeys preferred and avoided some fruit and arthropod items available in their home range, choosing a wide variety of arthropods. Geometrid moths (Lepidoptera) were the most important and consistent insects eaten over time. We found no differences in the type of arthropods adults and juveniles ate, but adults invested more time foraging for this resource, especially in moss. Although woolly monkeys are generalist foragers, they do not select their food items randomly or opportunistically.

## Introduction

Primates are characterized by having a broad range of foraging strategies and dietary preferences that are influenced by conservative factors such as morphology, behaviour, ecology and others^1^. Optimal Foraging Theory^2^ suggests that animals will use different behavioural and morphological strategies that will help them obtain preferred foods, that help them maximize nutrient intake at the lowest costs possible^3,4^. This theory also predicts that under low resource availability, generalists diet selection should broaden, to include fall-back foods in the diet, items of low nutritional value eaten particularly in periods of scarcity^5–8^. Primate foraging strategies are a response to spatial and temporal factors of resource availability, distribution and quality^9^. Nonetheless, characterizing these strategies and obtaining detailed information for generalist species can be very challenging, if not impossible, limiting our understanding of how primates change their foraging patterns in response to resource availability, nutritional demands and energy requirements^10^.

Primate species tend to be selective in what they choose to eat, often relying mainly on certain species and plant parts^9^. Indeed, they must select among resources to obtain proteins, fats, carbohydrates and essential compounds to fulfil their nutritional requirements^3,6^. Overall, dietary preferences do not only reflect nutritional needs and the availability of items but also behavioural flexibilities in how species compete for or prioritize foods, and might explain niche overlap between species exploiting the same resources^7,8^. For example, primate dietary choices are usually associated with Kay’s Threshold, which relates body size to diet and states that small insectivorous primates below 500 g should obtain protein from insects, and large primates mainly from plant material^11^. Primates from Central and South America seem to follow this hypothesis as small and medium body-sized monkeys like the San Martin titi (*Plecturocebus oenanthe*) and the white-faced capuchins (*Cebus capucinus*) obtain proteins from insects compared to larger primates that eat plants to get protein such as spider monkeys (*Ateles hybridus*) or howler monkeys (*Alouatta caraya*)^12–15^. Woolly monkeys however are an important exception to ‘Kay’s Threshold’. They are one of the largest Neotropical monkeys, and invest a lot of time in foraging for insects, although fruits are the main component of their diet^16,17^. This behaviour suggests that arthropods may have an important nutritional value for this monkey species, as they do for other smaller primate species^5,18–20^.

Woolly monkeys (*Lagothrix lagotricha*) are one of the largest primates in South America. Their diet consists mainly of ripe fruits^21–23^ and is complemented with arthropods, young leaves, unripe fruits, seeds, flowers^23–26^ and rarely small vertebrates^22,23^. Field observations and morphological identification from the remaining exoskeletons of insects in scats or stomach contents showed, that woolly monkeys eat a variety of arthropods such as beetles, crickets, fig wasps, leafhoppers, mantids, ants and spiders^23–25,27–29^. Further, in lowland populations, woolly monkeys forage for arthropods in substrates such as leaves, dead branches and spider webs^23^, while Colombian highland woolly monkeys at Cueva de los Guácharos forage actively on epiphytic substrates (i.e. moss and bromeliads), that in Andean and sub-Andean forests, harbour a high number of arthropods and hold a constant resource through time^16,30^. This might explain why up to 39% of the diet of woolly monkeys from a highland population is made up of arthropods^17^. Although dietary flexibility has been broadly examined by observational studies in woolly monkeys^11,20,21^, the lack of suitable methods to detect the sorts of arthropods they eat, and the challenges of obtaining detailed dietary records and evaluating complex social and environmental dynamics, have led to a superficial understanding of the feeding ecology of this primate species^31^.

Indeed, social dynamics can influence foraging patterns that can lead to competition among individuals of a group, including adults and juveniles, and might affect the amount of each dietary item they can obtain^32^. For example, in the case of lowland woolly monkeys at Tinigua National Park, these monkeys have a cohesive social structure, which implies that submissive age/sex classes, such as juveniles and non-lactating females, might spend more time feeding on insects than males as they are displaced from fruiting trees^23^. Further, sensorimotor abilities, social dynamics and foraging behaviour might also influence the ability of individuals to capture prey^33,34^. This “needing to learn” hypothesis states there should be differences between the places juveniles and adults forage and the sort of arthropods they are able to capture and eat^20,35^. Suggesting infants and young juveniles should miss more than adults when foraging for arthropods as these strategies might develop with age and practice. However, assessing these differences only through observational methods is quite challenging because obtaining visual records of the arthropod prey obtained by foraging monkeys is almost impossible or has potentially many biases.

Novel techniques, such as metabarcoding, the use of genetic barcodes, polymerase chain reaction (PCR) and high-throughput sequencing (HTS)^36,37^, have helped to address the predator–prey dynamics that are sometimes difficult to observe and quantify in the field^20,38–40^. Metabarcoding is a non-invasive technique that identifies the multiple taxa that are present in environmental samples (e.g. water, soil, scats) by amplifying short conserved gene sequences and running a parallel sequencing of the PCR amplicons^36^. Metabarcoding has been crucial in unveiling the feeding ecology, food webs, host-microbiota relationships and trophic interactions of all sorts of cryptic and elusive organisms^40–45^.

Here, we show how resource availability (i.e. fruits and arthropods) influences the feeding strategies of woolly monkeys and their dietary composition at the Parque Nacional Natural Cueva de los Guácharos, Huila, Colombia. For this purpose, we evaluated (i) the overall activity budget and diet composition of the Colombian woolly monkey, (ii) differences in the consumption of fruits and arthropods between adult and juvenile woolly monkeys, (iii) preferences and avoidances of fruits and arthropods over time, (iv) the relationship between food availability and feeding time in the canopy, and (v) using observations and metabarcoding, which fruits and arthropods are present in the diet of woolly monkeys over time.

## Materials and methods

### Study site

The study was carried out for 11 months from January to December 2018, with the exception of the month of August, in Cueva de Los Guácharos National Park, a 9000-ha protected area on the southwestern side of the Cordillera Oriental in the Colombian Andes (1°37′56″ N, 76°06′10″ W). The park contains mostly sub-Andean forests but also includes Andean and sub-páramo vegetation^46^. It has an annual mean rainfall of 2200 mm, with a dry period from December to February (average rainfall per month: 80 mm) and a rainy period from March to November (average rainfall per month: 234 mm). The elevation at the study site ranges from 1630 and 2850 m, though the park reaches up to 3000 m. The home range of the main study group (472 ha) included sub-Andean pristine and old secondary forests located between 1800 and 2747 m above sea level^17,47^.

### Behavioural observations

Colombian highland woolly monkeys (*Lagothrix lagothricha lugens*) weigh 8–10 kg and are one of the largest primates of South America. This primate is considered an endemic subspecies categorized as Critically Endangered (CR) because of hunting, habitat destruction and illegal capture for the pet trade. We collected behavioural data of two habituated groups (“Colombia”, N = 34, 4 males, 8 females, 2 subadult males, 1 subadult female and 19 juveniles) (“Brasil”, N = 33, 8 males, 9 females and 16 juveniles) and a new non-habituated group (“Chile”, N = 25, 6 male adults, 5 females and 14 juveniles) of Colombian woolly monkeys *Lagothrix lagotricha lugens* that overlapped in their home ranges. We collected focal time samples on individuals that were within 3–15 m of the observer from dawn to dusk (6:00 to 18:00)^48^. Most of the monkeys of “Colombia” and “Brasil” and a few of “Chile” were recognizable by facial features, body size or natural marks on the genitalia. We followed each focal animal for a short period of time prior to the identification of the individual or it’s sex and age class if the identity was unknown. Then we recorded its behavioural state (e.g., moving, resting, feeding and social interactions) every 10 min for a minimum of 2 h and if possible, we obtained a scat sample from that focal animal before changing to another focal individual. If an individual was out of view for 20 min we changed to a new focal animal. We were able to record a total of 416 h of focal time sampling.

Subadults (N = 3) and juveniles’ data were grouped in the same category for future analyses because of the low number of subadults in the population. When the focal animal was feeding, we noted the type of food it was eating (i.e., ripe fruit, unripe fruit, young leaves, flowers, seeds or arthropods) and where it was being obtained from (i.e. moss, bromeliads, flowers, branches). When eating arthropods, we recorded if the focal animal was gripping and tearing apart bromeliads, inspecting substrates, licking leaves and trying to capture flying arthropods or if they had their hands closed and moved them towards the mouth when they managed to capture non-flying arthropods. We did not include arthropods inadvertently ingested in infested fruits^49^.

### Arthropod abundance

Arthropod abundance in the canopy was simultaneously measured using two complementary techniques. First, we installed 12 modified composite traps for aerial insects, hanging from an average height of 15 m above ground once a month for 168 h (1 week). Each month we randomly changed the location of the composite traps to avoid pseudoreplication^50^. The composite trap^51^ consists of the union of three traps normally used to capture insects (Malayse, Pitfall and Interference trap). The trap contains two receptacles, one at the bottom filled with soapy water where arthropods will sink and one empty receptacle at the top that insects cannot escape from because of their positive phototropism. Each aerial composite trap was distanced at least 500 m from the next one in both pristine and secondary forest of the woolly monkeys’ home range.

We also climbed up to the canopy (from 5 up to 21 m) to collect arthropods manually using straps on the legs, a harness and a sling. Straps were wrapped around the tree trunk to create foot loops for the legs, securing a lanyard to the harness, and climbing the tree by pulling up the webbings one by one. We collected the arthropods monthly from two different substrates (mosses and bromeliads) over 10 months using a branch bagging and clipping method^52^ from lower to higher heights following canopy stratification^53^. This technique involves passing a mesh or cloth bag over a portion of the substrate, then removing it from the tree and immediately placing it in a bag. We identified the arthropods to the taxonomic level of family and divided them into morpho-species, and these were then dried in absorbing paper and at 45 °C to constant mass at Universidad de Los Andes^52,54^.

In a previous study at Cueva de Los Guácharos we did not find a significant relationship between the arthropod biomass among tree species^16^, for that reason we randomly selected 20 trees inside the home range of the study group regardless of their species—*Saurauia brachybotrys* (Actinidiaceae), *Tapirira guianensis subandina* (Anacardiaceae), *Hedyosmum cuatrecazanum* (Chlorantaceae), *Alchornea grandis* (Euphorbiaceae), *Incadendron esseri* × 2 (Euphorbiaceae), *Sapium* cf*. cuatrecasasii* (Euphorbiaceae), *Inga oerstediana* (Fabaceae), *Quercus humboldtii* (Fagaceae), *Juglans neotropica* (Juglandaceae), *Nectandra* sp. × 2 (Lauraceae), *Nectandra purpurea* (Lauraceae), *Heliocarpus americanus* × 2 (Malvaceae), *Blakea calyptrata* (Melastomataceae), *Miconia minuta* (Melastomataceae), *Morus insignis* (Moraceae), *Hyeronima huilensis* (Phyllanthaceae), *Allophylus* cf*. punctatus* (Sapindaceae)—which were distanced 250 m apart and with the restriction that the DBH was not greater than 300 cm, as trees with DBH larger than 300 cm were not possible to climb with this technique.

We collected moss (N = 183) and bromeliads (N = 164) in each ascent for every selected tree; each bromeliad was completely destroyed in order to collect all arthropods within the sample. Bromeliads were not discriminated by size, species, diameter, or leaf-base structure when collected. However, we evaluated the arthropod biomass as arthropods dry weight divided by the bromeliads and moss weight^52,54^. As aerial composite traps and substrate biomass had different and not comparable sample units, we tested if arthropod biomass was related to substrate weight. In both, moss (Pearson: t = − 1.53, df = 8, P = 0.16) and bromeliads (Pearson: t = − 0.35, df = 8, P = 0.73) we found weak correlations between these variables, suggesting the abundance of arthropods inside a substrate sample is independent of its size. For that reason, for further analyses arthropod abundance of aerial composite traps and substrates was quantified as the logarithm of the arthropod abundance.

### Fruit productivity

Forest-wide fruit productivity was estimated over 11 months using 168 fruit hanging traps^55,56^ in four transects inside the home range of the study groups. Each trap consisted of a 0.64 m^2^ cloth tied up with nylon to neighbouring trees and was distanced 25 m from the next trap and randomly located between 0 to 10 m perpendicular to the path. We collected the content of the traps in individual, labelled plastic bags twice a month. We separated from each bag all plant material (i.e. leaves, flowers and fruits) and distributed each of them separately in labelled paper bags with the respective trap they were originally collected from. Flowers and ripe fruits were identified to the taxonomic level of species and only the latter was used for statistical analyses. Afterwards, all the fleshy ripe fruit bags were dried in an oven and weighed individually for each species present in each paper bag in order to quantify fruit productivity in terms of kg/ha^57^.

### Collection of dietary samples

Between February and December 2018, we collected 247 fecal samples from adults (60%) and juveniles (40%) from the three different groups of woolly monkeys. In order to minimize exogenous contamination from eDNA, fecal material was collected as soon as possible after defecation into 2 mL Eppendorf’s with sterile skewers. Samples were obtained from mostly the inner part of the scats and sides of the samples in direct contact with the floor or litter were removed before collecting the sample to avoid cross-contamination. RNALater was added with a volume ratio of 1:1 to avoid DNA degradation and enzymatic activity. Each sample was registered with a serial code, age (i.e. adult, juvenile), sex, time and date. Samples were kept in the dark and at room temperature (15 °C) until they could be stored at − 20 °C^58^ at Laboratorio de Ecología de Bosques Tropicales y Primatología (LEBTYP) at Universidad de los Andes, Colombia.

### DNA extraction and PCR amplification

DNA was extracted from all 247 samples using the NucleoSpin DNA Stool Mini Kit (Macherey-Nagel) following the manufacturer instructions. DNA concentration was measured using Nanodrop, samples with a yield below 10 ng/μL were discarded. Afterwards, we prepared pooled samples divided into adults and juveniles for each month of sampling (N = 60). Because pooling might reduce the molecular sequence richness, as less frequent and rare prey DNA is obscured by more abundant DNA^59^, for each month and age we prepared two biological replicates in order to be able to detect less common prey within the samples^40^.

We used a DNA barcoding approach to detect arthropods ingested by woolly monkeys. We amplified two universal highly degenerated arthropod primer for the cytochrome oxidase I (*COI*) mitochondrial gene: ZBJ (~ 157 bp) ArtF1c: 5′ AGATATTGGAACWTTATATTTTATTTTTGG 3′; ArtR2c: 5′ WACTAATCAATTWCCAAATCCTCC 3′^60^ and ANML (~ 180 bp) F: 5′ GGTCAACAAATCATAAAGATATTGG 3′; R: 5′ GGWACTAATCAATTTCCAAATCC 3′^61^. Both primers were amplified via a one-step PCR with tagged primers. This approach simplifies the efficiency of the amplification and prevents the high occurrence of tag jumps prior to High-Throughput Sequencing (HTS)^42,62,63^.

We prepared a PCR master mix for each sort of sample with a total volume of 20 μL included: 10 μL of Platinum II Hot-Start PCR Master Mix (Invitrogen), 0.8 μL of 10 nM F primer, 0.8 μL of 10 nM R primer, 7.4 μL of nuclease-free water and 1 μL of DNA. For each sample we amplified three technical replicates, as this might also increase the odds of detecting rare prey within the samples^63^. DNA was amplified using the following Access Array cycling: 5 min at 95 °C, followed by 30 cycles of 94 °C, 1 min at 45 °C, 30 s at 72 °C, followed by a final extension step of 72 °C for 5 min. PCR products were visualized on 2% agarose gels and purified using QIAquick PCR Purification Kit (QIAGEN) following the manufacturers protocol.

### Metabarcoding sequencing analysis

The library was built and sequenced at the Illumina MiSeq Nano v_2_ (2 × 150) platform at Zentrum für Quantitative Biologie (QBiC) (Tübingen, Germany). We included negative and positive controls among the sequenced samples^63^. Sequences were demultiplexed, primers and adapters were removed from the sequences with the *cutadapt* tool. Sequence reads were processed using the DADA2 *v1.18.0* package^64^. Samples were joined via PE, dereplicated, and amplicon sequence variants (ASVs) were inferred by pseudo-pooling instead of inferring operational taxonomic units (OTUs), because of its much higher resolution identifying single base differences and targeting real unique sequences^65^. Chimeric sequences were identified and removed. Afterwards, ASVs were assigned taxonomy using the built-in curated COI barcode of life database (BOLD) with the *AMPtk v 1.4.0* tool^66,67^. ASVs that matched with negative and positive controls, as a result of tag jumpings during the sequencing^63^, and sequences with match identity below 90% were removed manually.

Metabarcoding is perhaps the most efficient technique for describing the arthropod feeding ecology of this primate species, compared to for example, direct observations and items remaining in the scats^38,39,68^. Nevertheless, this method still has some disadvantages^69–71^. One of the most evident is that databases, genes and markers have biases assigning taxonomy, as there are over-represented invertebrate orders with DNA barcodes in the nucleotide databases^70,72–75^. Another relevant bias, especially in the Americas, is the lack of barcodes for regional species. Despite these limitations, we decided to use this approach as it is still possible to have a quite good resolution of the different taxa present in the samples.

### Statistical analyses

All statistical analyses were done and figures made using R Studio *version 4.1.2*^76^ with the *nlme*, *raster*, *sp*, *dada2*, *vegan*, *ggplot2* packages^44,61,64,77,78^. We obtained precipitation data from the free data server *worldclim* (https://www.worldclim.org) and average monthly values were obtained for Cueva de Los Guácharos coordinates. We ran two linear mixed models, one to test the effects of arthropod availability in the canopy on the feeding time over the duration of the study, and another to test the preferences among arthropod orders present in the woolly monkeys diet over time. Time was set as a random effect to control monthly for pseudoreplication, while the independent variables of substrate type and arthropod type were included as fixed effects in their respective tests. We used linear models to test the relationship between resource availability and the feeding time monkeys spent eating each item, to evaluate the effect of precipitation on arthropod availability in different substrates, and the relationship between arthropod availability and arthropods present in the diet. We used ANOVAs to test for differences between age classes in arthropod consumption, as well as for testing for differences in the abundance of arthropods among substrates and predation on different types of arthropods. We used Jacobs Selectivity Index^40^, which tests the predator’s preference to prey based on relative abundance of consumed prey and the relative abundance in the environment, to evaluate preferences for any sort of arthropod and fruit in the diet related to the availability of each resource in the canopy. We used an alpha threshold value of 0.05 to test our hypotheses.

### Statement of ethics

We obtained permits from Parques Nacionales Naturales de Colombia and Universidad de los Andes to conduct this study. This project adhered to all legal requirements necessary under Colombian laws. We did not have any direct interactions with woolly monkeys that could affect their well-being and samples from woolly monkeys were collected via non-invasive methods as described above.

## Results

**Figure 1:**
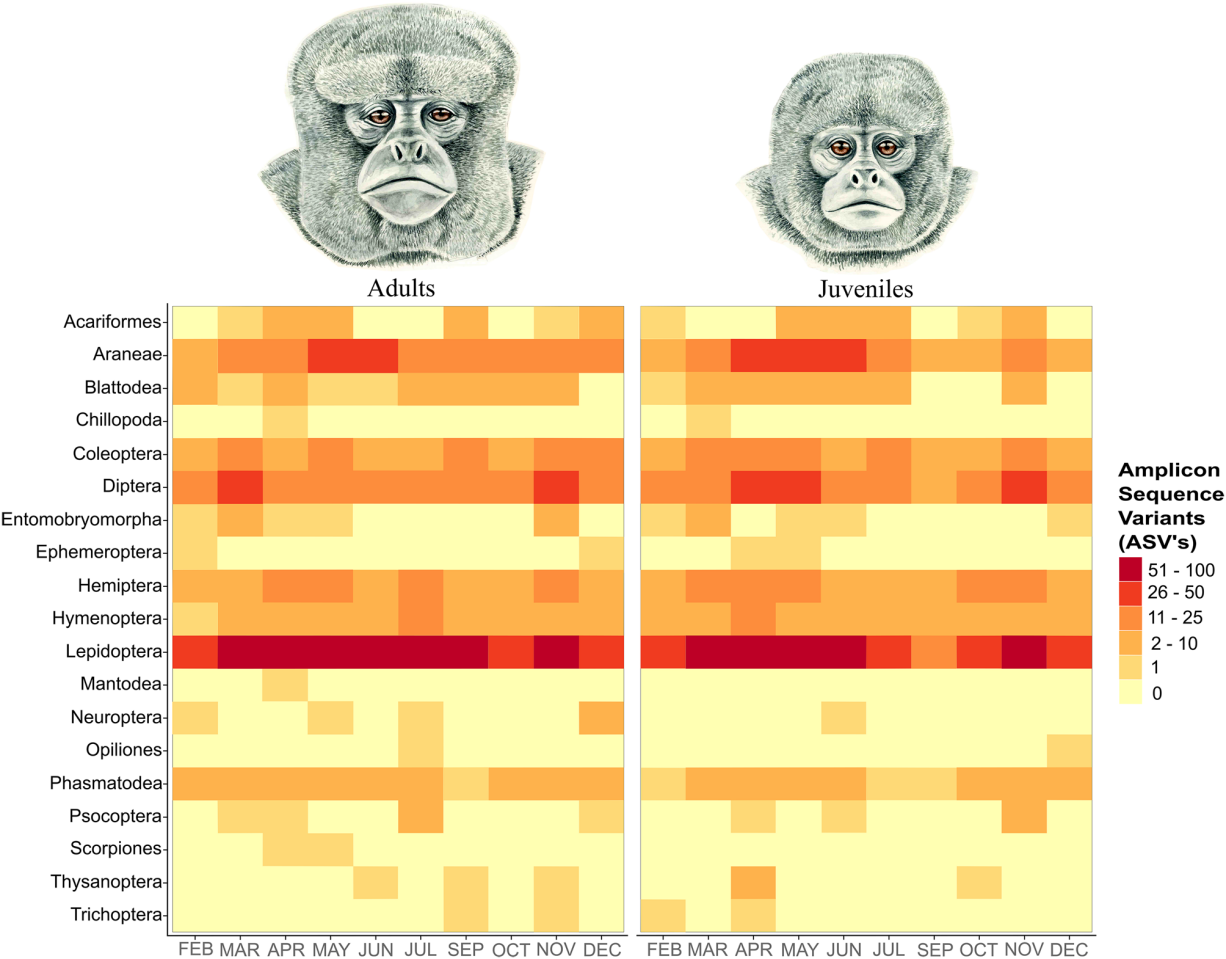
Arthropod consumption differences between adult and juvenile woolly monkeys (*Lagothrix lagotricha lugens*) at Cueva de los Guácharos National Park. Amplicon Sequence Variants (ASV’s) counts for each order of arthropod eaten by adult and juvenile woolly monkeys over the study period. Regardless of age class, woolly monkeys seem to eat in general the same types of arthropods and during the same periods of time. The heatmap indicates that the most eaten and selected arthropod for adults and juveniles are Lepidopterans. Light colours indicate low availability of ASV’s while darker colours indicate a high amount of ASV’s present in the woolly monkeys’ scats. Woolly monkeys artwork was painted by the biologist and artist Angela Mejía.

Colombian highland woolly monkeys invested most of their time in feeding (44%), moving (29%), resting (23%), social activities (2%), and others (2%). The most consumed items during the study were fruits (54%), followed by arthropods (28%), young leaves (14%), flowers (3%) and seeds (1%). The age class of individuals did not have a significant impact on the feeding time of fruits (ANOVA: F = 2.93, df = 18, P = 0.10). Ripe fruit productivity did not predict the fruit feeding time of the monkeys (Pearson: t = − 0.69, df = 8, P = 0.51). On the contrary, we did find a significant relationship between age class and feeding time on arthropods (ANOVA: F = 4.80, df = 18, P = 0.04), with adults eating arthropods more than juveniles, but not in the types of arthropods that adults and juveniles ate (ANOVA: F = 0.24, df = 198, P = 0.62) (Fig. 1). We found a difference between the arthropod abundance in the canopy and feeding time on arthropod prey (Pearson: t = 2.53, df = 8, P = 0.03). Further, we found a positive relationship between the time highland woolly monkeys feed on fruits and arthropods (Pearson: t = 2.74, df = 8, P = 0.02).

**Figure 2:**
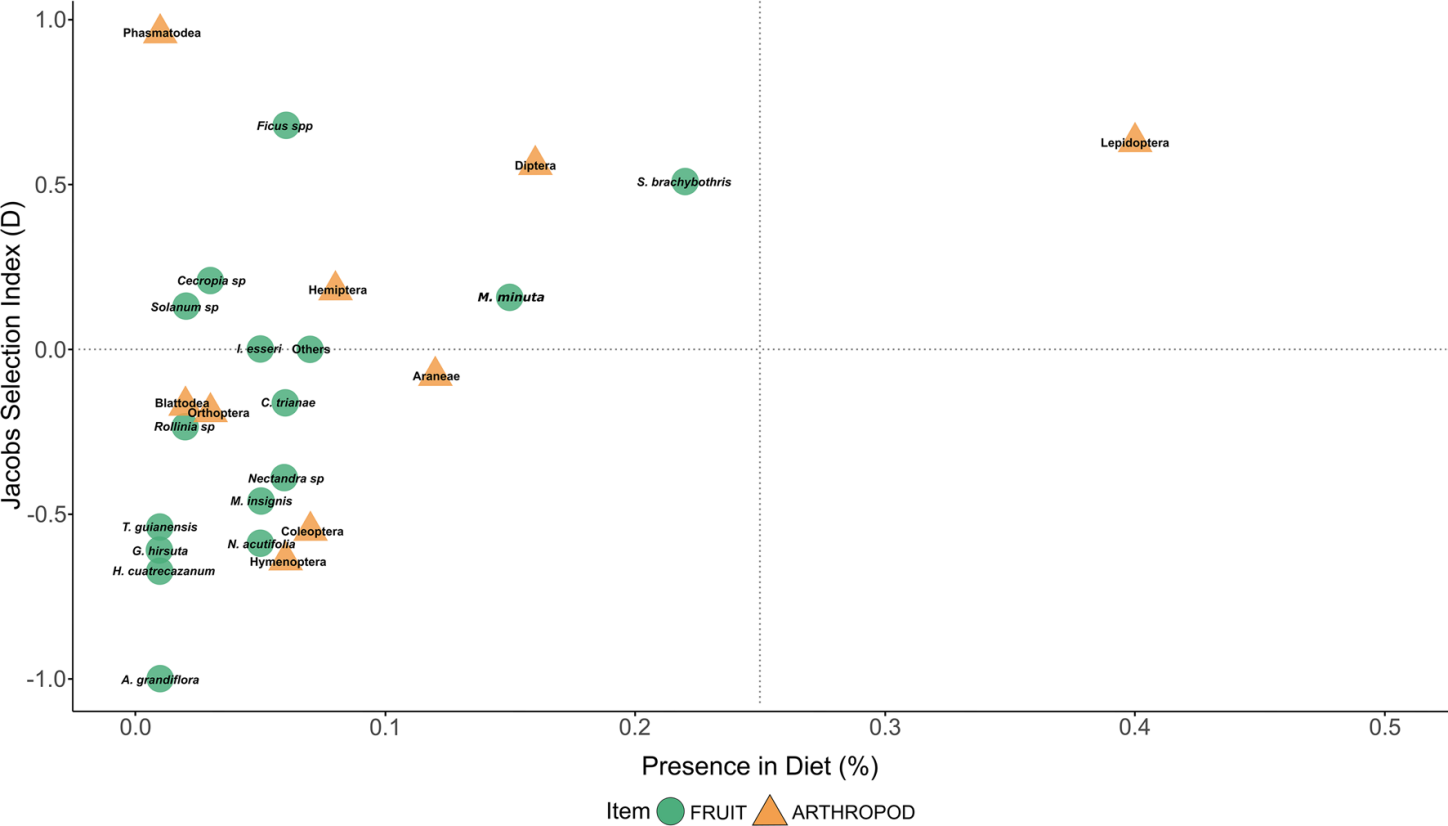
Fruit and arthropod preferences by Colombian highland woolly monkeys (*Lagothrix lagotricha lugens*) at Cueva de los Guácharos National Park. Results show the dietary preferences of fruits (green circles) and arthropods (orange triangles), y-axis shows how much an item is eaten compared to its availability, Jacobs’ selectivity index (D), and compared to x-axis how much each item is present in the monkeys diet (%). Items with higher values than 0.5 are the most selected, while below − 0.5 are among the most avoided by the monkeys.

**Figure 3:**
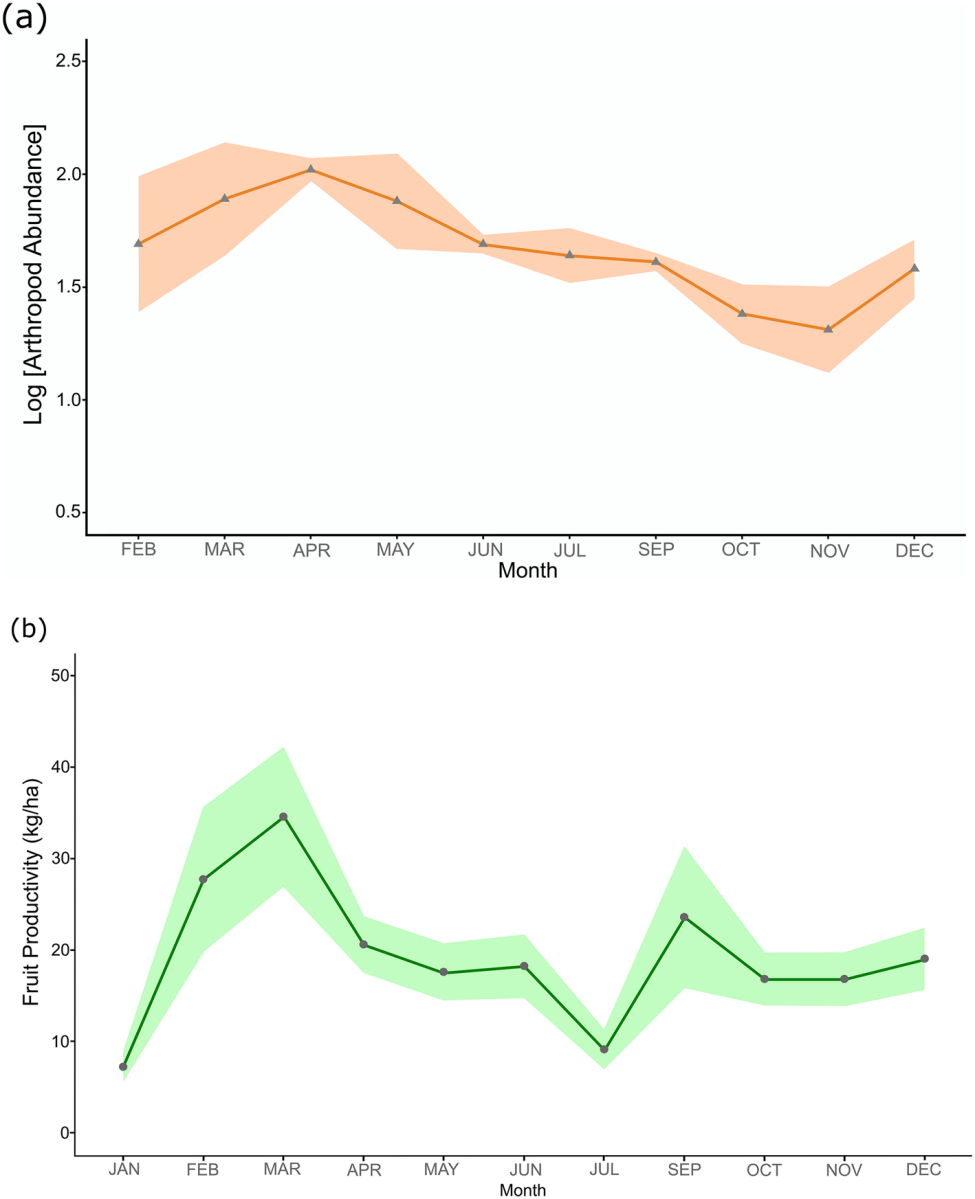
Arthropod and fruit availability at Cueva de los Guácharos National Park. (**a**) Arthropod abundance (orange) over time. In general, availability is quite constant over time and peaks during the first months of the year, experiencing a decrease during the last months of the study. Light orange shadow around each point indicates the standard error. (**b**) Seasonal variation of fruits (green) over time, y-axis shows the mean month fruit productivity in terms of kg/ha. Productivity peaks twice a year during the months of March and September, light green shadow indicates the standard error.

We recorded Colombian woolly monkeys eating from at least 35 plant species, for which we did find differences in the selection of fruits items among species (ANOVA: F = 3.54, df = 171, P < 0.001) (Fig. 2). Woolly monkeys favoured eating fruits of species like *Saurauia brachybotrys, Ficus *spp.,* Miconia minuta*, and also were indifferent or avoided species such as *Alchornea grandiflora, Nectandra acutifolia* and *Tapirira guianensis subandina* compared to their availability (Table 1). We found preferences in the type of arthropods woolly monkeys ate over time controlled by the order of arthropod as a fixed effect (Mixed Model: F = 7.94, df = 9, P = 0.002). Lepidopterans were the most selected and consistent arthropod present in the diet (Fig. 3b). Further, we found woolly monkeys prefer orders of arthropods such as Lepidopterans, Dipterans, Hemipterans and Phasmids and avoid orders such as Hymenopterans and Coleopterans (ANOVA: F = 14.41, df = 90, P < 0.001) (Fig. 2).

**Table 1:** Top fruiting species productivity and how much is included in the woolly monkeys’ (*Lagothrix lagotricha lugens*) diet during the study period at Cueva de los Guácharos National Park. Fruiting species with an asterisk (*) point out that were present in the diet of the monkeys but we were not able to detect them in our fruit hanging traps.

Rank	Species	Family	Productivity (kg/ha)	% in diet
1	*Inga exalata*	Fabaceae	45.65	2.77
2	*Nectandra *sp.	Lauraceae	38.29	7.38
3	*Cissus trianae*	Vitaceae	36.05	6.46
4	*Alchornea grandiflora*	Euphorbiaceae	25.28	0.92
5	*Nectandra acutifolia*	Lauraceae	24.64	5.23
6	*Saurauia brachybotrys*	Actinidiaceae	21.55	22.46
7	*Tapirira guianensis subandina*	Anacardiaceae	19.95	1.23
8	*Guatteria hirsuta*	Annonaceae	12.27	1.23
9	*Incadendron esseri*	Euphorbiaceae	10.77	5.23
10	*Hedyosmum cuatrecazanum*	Chloranthaceae	9.81	0.92
11	*Miconia minuta*	Melastomataceae	9.17	15.38
12	*Vismia mandur*	Hypericaceae	8.64	0.92
13	*Guatteria goudotiana*	Annonaceae	7.45	0.62
14	*Blakea *sp.	Melastomataceae	6.61	0.62
15	*Oreopanax cheirophylus*	Araliaceae	6.29	1.23
16	*Morus insignis*	Moraceae	4.27	4.62
17	*Ficus *spp.	Moraceae	3.2	6.46
18	*Croton magdalensis*	Euphorbiaceae	2.13	1.54
19	*Helicostylis tovarensis*	Moraceae	1.81	0.62
20	*Sapium stylare*	Euphorbiaceae	0.23	2.46
21	*Solanum *sp.	Solanaceae	0.13	2.46
22	*Cecropia *sp.	Urticaceae	0.00*	3.08
23	*Rollinia *sp.	Annonaceae	0.00*	2.15
24	Others		95.43	4.00
		Total	389.62	100

We collected a total of 1901 arthropods. These were classified into 22 orders and 111 families of insects, arachnids and terrestrial crustaceans (order: Isopoda). The arthropod community at Cueva de los Guácharos appears to be equally distributed among the different substrates (Fig. 3a). We identified among all substrates combined a high number of ants (Hymenoptera: Formicidae, N = 253), scaffold web spiders (Araneae: Nesticidae, N = 137), geometer moths (Lepidoptera: Geometridae, N = 67), leaf beetles (Coleoptera: Chrysomelidae, N = 42), leafhoppers (Hemiptera: Cicadellidae, N = 37) and striped earwigs (Dermaptera: Labiduridae, N = 28).

**Figure 4:**
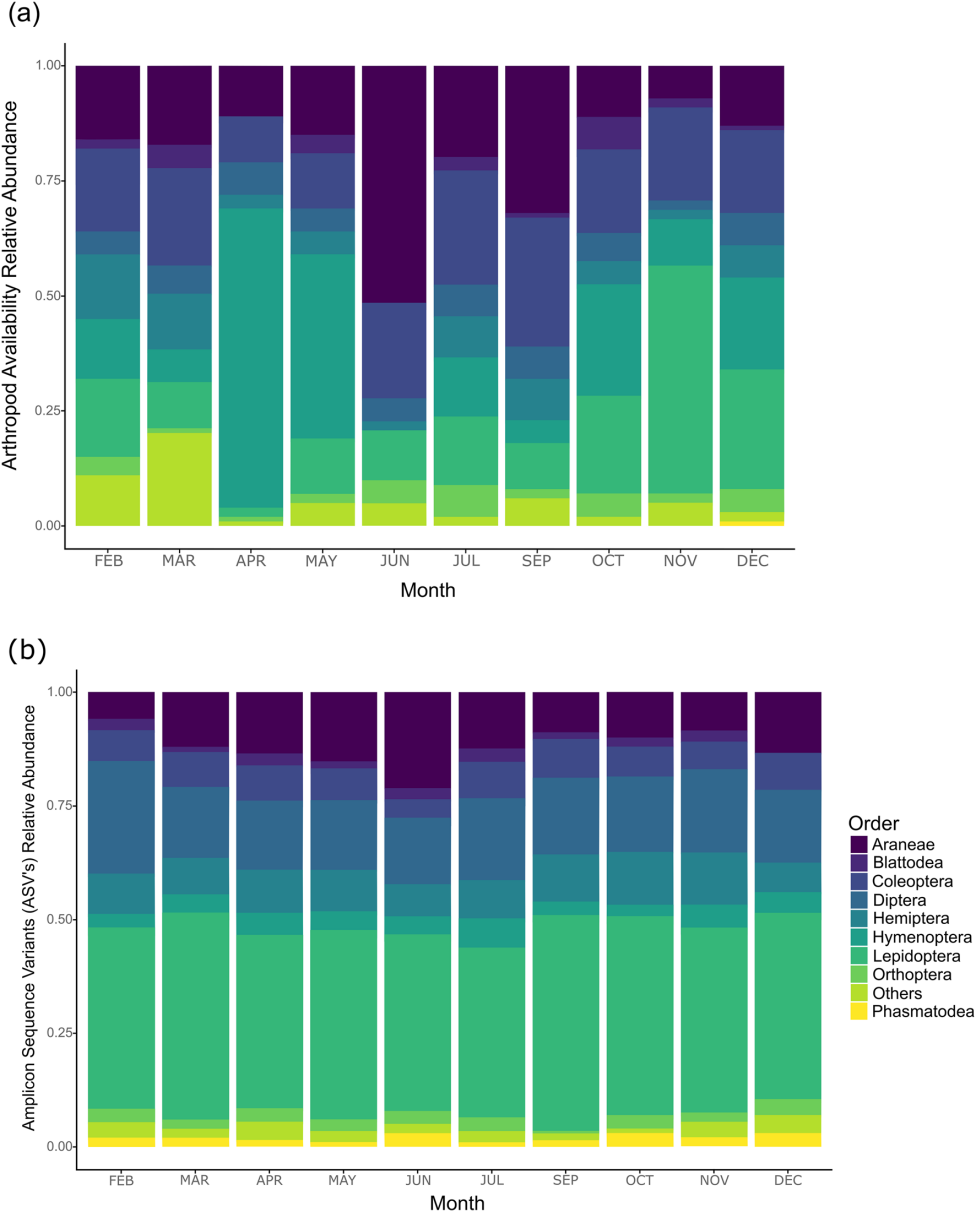
Arthropod abundance and consumption comparison by Colombian highland woolly monkeys (*Lagothrix lagotricha lugens*) at Cueva de los Guácharos National Park. (**a**) Results of relative arthropod abundance show the changes of different orders of arthropods during the study. Changes in composition and abundance of arthropods at Cueva de los Guácharos National Park, shows how constant humidity can harbour arthropods all over the year. (**b**) Relative abundance of arthropods Amplicon Sequence Variants (ASV) present in the woolly monkeys’ scats. Results show a constant preference during the entire study for Lepidopterans (i.e. geometer moths) and few variations in the consumption of the most relevant arthropods during the study.

We did not find significant variation in arthropod availability in the canopy over time controlled by the type of substrate as a fixed effect (Mixed Model: F = 1.19, df = 9, P = 0.36). Still, arthropods increased between March and May and decreased considerably during October and November (Fig. 4a). We did not find differences in the number of arthropods that could be found among substrates (ANOVA: F = 1.19, df = 27, P = 0.32). Nonetheless, 72% of the arthropod foraging events were registered in moss, the substrate where the monkeys invested the most time in foraging, in comparison with bromeliads, leaves or flowers (ANOVA: F = 7.63, df = 36, P < 0.001). Arthropod availability was not related with the monthly precipitation in any of the studied substrates: bromeliad (Pearson: t = 1.43, df = 8, P = 0.19), canopy (Pearson: t = 1.87, df = 8, P = 0.09) and moss (Pearson: t = 1.95, df = 8, P = 0.08).

We registered 90 plant species producing ripe fleshy fruits on the hanging traps. The total annual forest fruit productivity was 389.62 kg/ha. The most productive species were *Inga exalata, Nectandra *sp.,* Cissus trianae, Chamaedorea linearis* and *Saurauia brachybotrys*. We observed woolly monkeys feeding on these productive tree species except for the understory palm (*Chamaedorea linearis*). We found fruit productivity peaks two times in the year, one during February–March (e.g. *Guatteria hirsuta, Saurauia brachybotrys*) and the second one during September (e.g. *Cissus trianae*) (Fig. 4b).

We identified a total of 2483 unique sequences in woolly monkey scats. 1191 ASVs were assigned for the ZBJ marker with an average of 28k reads per sample, and only 16 sequences were removed after contrasting with the HTS controls. We found 1292 ASVs for the ANML marker with an average of 12k reads per sample, and 75 sequences were removed from the negative controls after HTS. Overall, we identified 23 ASV to the order level (97%), 182 to the family level (64.24%), 490 to the genus level (47.30%) and 198 to the species level (12.49%). for which 70% of the ASVs were present in both markers. The most abundant sequences were from Lepidopterans (40.15%), Dipterans (16.35%), Araneae (11.37%), Hemipterans (7.5%) and Coleopterans (6.92%) (Table 2).

**Table 2:** Arthropod Amplicon Sequence Variants present in the scats of Colombian highland Woolly Monkeys (*Lagothrix lagotricha lugens*) at Cueva de los Guácharos National Park.

Class	Order	ASV counts	Relative abundance	% in diet
Insecta	Lepidoptera	685	0.402	40.15
Insecta	Diptera	279	0.164	16.35
Arachnida	Araneae	194	0.114	11.37
Insecta	Hemiptera	128	0.075	7.50
Insecta	Coleoptera	118	0.069	6.92
Insecta	Hymenoptera	98	0.057	5.74
Insecta	Orthoptera	46	0.027	2.70
Insecta	Blattodea	29	0.017	1.70
Insecta	Phasmatodea	17	0.010	1.00
Arachnida	Trombidiformes	16	0.009	0.94
Arachnida	Sarcoptiformes	7	0.004	0.41
Insecta	Neuroptera	5	0.003	0.29
Insecta	Tichoptera	4	0.002	0.23
Insecta	Ephemeroptera	3	0.002	0.18
Arachnida	Scorpiones	1	0.001	0.06
Insecta	Mantodea	1	0.001	0.06
Others	No ID	75	0.044	4.40
	Total	1706	1	100

## Discussion

Colombian highland woolly monkeys select some resources more often than others compared to their availability in their wild habitat. Energy has been suggested to be the primary driver behind food choice^79^, and consequently, Atelid primates have been observed to prefer primarily fruits rich in lipids and carbohydrates^3,4,22,23^. This might explain why the most important food item for the highland woolly monkeys were fruits (54%) and is consistent with previous reports for woolly monkeys at Guácharos and other locations: Yasuní: 76.7%, Guácharos: 60%, Tinigua: 60%^16,22,23^. However, woolly monkeys’ fruit selection seemed to vary between seasons and is more flexible during periods of scarcity, as despite feeding from the most productive species, they included a considerable amount of fall-back foods (e.g. *Ficus *spp., unripe fruits) in their diet.

Woolly monkeys at Cueva de los Guácharos included a great variety of arthropods in their diet, with this food source representing up to 37% of the feeding records in periods of high abundance. Our results are consistent with previous reports from the same population at Guácharos: 39%, 39.5%^16,17^, highlighting insects and arachnids as a crucial source of protein and micronutrients^5,18^. Unfortunately, researchers have not focused on the contributions of fruits and arthropods simultaneously in the diet of primate species^20^. Mostly because feeding ecology studies concentrate either on fruits or arthropods separately due to the difficulties of assessing, collecting, processing and identifying both items at the same time^5,80^.

Balancing carbohydrate and protein macronutrients might influence food choices considerably as primates must fulfil their daily nutritional requirements and this could be considered as a behavioural strategy to maximize nutrient intake compared to investing more foraging for only a particular item^10,81^. For example, we found that fruit and arthropod feeding time peaked during the same periods of time, thus there is a positive relationship between eating both resources. This positive relationship suggests these resources might complement and balance this primates’ diet, as together they would provide better nutrition than separately as both resources bring different and important nutrients to the diet^22,23^, or on the contrary this could be coincidental, as fruit and arthropod abundance peak at the same period of time increasing the availability of both resources and perhaps as a consequence woolly monkeys consume both. However, feeding time is not an exact measure and it might still have biases, as for example the number of items a monkey can eat per unit of time, or individual preferences monkey’s might have for certain food items. Future studies should consider how time investment on a resource can influence nutritional gain, a real measure of what a resource brings to the diet, and for which it is very challenging to obtain data. Ideally, physiology, morphological traits and weight variation through time should be included in feeding ecology studies.

Woolly monkeys are the only species from the Atelidae family that invest such a considerable amount of time foraging for arthropods. In contrast, southern muriquis (*Brachyteles arachnoides*), woolly monkey’s sister species, are as expected mainly frugivorous (75%), and to our knowledge arthropod consumption has not been reported so far^82,83^. Similarly, the highly frugivorous spider monkey (*Ateles hybridus*) rarely eats arthropods (1.5% in a study year)^14^. Finally, black-and-gold howler monkeys (*Alouatta caraya*) and black howler monkeys (*A. pigra*) eat lots of leaves to obtain the protein compounds they need, and insects have been detected in their diet only as a secondary predation effect of larvae infesting seeds that these monkeys feed from^12^ or ants from leaves and stems that have a mutualistic relationship with plants, such as *Cecropia *spp., that are part of their diet^84^. These comparisons highlight the importance of woolly monkeys as an exception to Kay’s threshold^11^, perhaps evidencing how consuming such an important number of arthropods could be a strategy to decrease competition with other sympatric frugivorous primate species (i.e. spider monkeys; *Ateles* spp.). Resource competition might have reinforced a directional selective pressure that influenced the development of morphological and behavioural traits in woolly monkeys^80,85^, that allowed them to exploit this resource, as it has also been observed for the partitioning of foraging strategies and diets of sympatric primate species from the Americas^9^.

Despite not finding major differences in the types of arthropods woolly monkeys ate, we did find that adult woolly monkeys devoted more time to foraging for arthropods than juveniles. This could be related to the fact that the groups of this population usually split into separate groups (fission–fusion) spreading out more than lowland woolly monkeys’ populations, reducing the competition for resources among group members^47^. Additionally, highland woolly monkeys from Cueva de Los Guácharos have lower aggression rates from male adults to other group members than in other populations^86^. Perhaps also explaining why there are no significant differences in the amount of fruit feeding time between adults and juveniles. However, these behavioural patterns contrast enormously with other woolly monkey populations as lowland populations have different social structures that reflect more intense competition for resources between age/sex individuals inside the same groups^23^.

Competition between adults and juveniles depend on the temporal and spatial distributions of resources^32^. In the case of lowland woolly monkeys at Tinigua National Park, these primates have a cohesive social structure, which suggests that submissive age/sex classes, such as juveniles and non-lactating females, spend more time feeding on insects than males as they are displaced from fruiting trees. Further, sensorimotor abilities, social dynamics and foraging behaviour might also influence the ability of individuals to capture prey^33,34^. Contrary to our expectations regarding the needing to learn hypothesis, adults and juvenile woolly monkeys ate in general the same types of arthropods. Implying that both have similar sensorimotor abilities and share arthropod foraging strategies to capture prey^20,35^. Additionally, social dynamics (i.e. grooming) show that regardless of age, woolly monkeys at Cueva de los Guácharos are able to identify and eat ectoparasites such as mites^33,34^. Nonetheless, it is important to highlight that only adults were able to eat invertebrates that are very difficult to capture such as mantids and scorpions, showing that probably over time, experience allows these monkeys to broaden their diet.

Woolly monkeys selected insects with impressive consistency; as shown by the avoidance of Hymenopterans such as venomous wasps and Coleopterans because of their hardened exoskeletons. Indeed, Hymenoptera are one of the most avoided insects in the study, perhaps because the monkeys avoid insects such as venomous wasps. It is curious that woolly monkeys at Cueva de los Guácharos did not eat a high number of ants compared to other populations^23,27,29^, especially as these invertebrates were indeed the most abundant insect in the canopy of Guácharos and usually exhibit a high abundance in cloud forest because of their symbiosis with tank bromeliads *Tillandsia *spp.^87,88^; perhaps this could be related to their preference for other insects such as moths, which are richer in terms of proteins and micronutrients^5,18^.

The preference for insects such as Lepidopterans (i.e. geometrid moths), Dipterans, Hemipterans and Phasmids reflect that despite being generalist foragers, woolly monkeys can select prey beyond opportunistic and random patterns. Although geometric moths were overall one of the most abundant arthropods in the study, which might be explained by the hypothesis that Andean rainforests elevations might act as biodiversity hotspots of animals such as insects, birds and vascular plants^89,90^, it is evident in the metabarcoding sequences that woolly monkeys forage for geometric moths even when other arthropods are more abundant and available in the canopy. Indeed, Andean cloud forests have a high diversity of moths and spiders, as well as a great number of newly discovered species in these unexplored regions^89,91,92^. We believe woolly monkeys are most likely able to find moths all over the canopy despite variables such as canopy strata, insect biological rhythms (e.g. diurnal, nocturnal), seasonality and dispersal.

Insects and other invertebrates can be considered high-quality foods. Specifically, compared to other arthropods, arthropods such as Lepidopterans include nutritionally a high component of proteins (42.5%) and a higher percentage of fats (51.4%)^5^. Nevertheless, obtaining these proteins and lipids is a big challenge as chitin exoskeletons are made of a polysaccharide very difficult to degrade^93^. In fact, we were not able to find any invertebrates’ parts remaining in the scats; this is quite a strange phenomenon as the monkeys include a large number of arthropods in their diets during the entire year. This suggests that woolly monkeys must have digestive strategies like chitinase enzymes or longer retention times that could facilitate the microbial fermentation and break down of chitin^94,95^. From our results, it is evident how much time the monkeys from this population invest in foraging for arthropods, which can be mainly attributed to a large number of epiphytic substrates in the canopy that can harbour a great number of arthropods^30,91^.

Colombian highland woolly monkeys at Cueva de los Guácharos foraged actively on epiphytic substrates (i.e. moss and bromeliads), that in Andean and sub-Andean forests harbour a high number of insects, arachnids and terrestrial crustaceans^16,30^. These tropical rainforests possess high humidity, allowing the relatively constant occurrence of arthropods over time^91,96^. Despite finding no differences in the number of arthropods that could be found among substrates, 72% of the arthropod foraging events were registered in moss, being by far the substrate the monkeys invested most time in foraging, in comparison with bromeliads, leaves or flowers. Even though from all sampled substrates mosses had the smallest size, the amount of moss in the canopy may outnumber spatially the other substrates and mosses can retain more water volume which would provide a suitable covert microhabitat for arthropods^85,97^.

Arthropod availability increased significantly at the beginning of the rainy season in March and May, peaking at the same period of time as ripe fruits did (March–April), and decreased considerably between the transition of rainy to dry season (October and November). Which a priori could be related to the beginning of rainfall as fruiting trees synchronize at this period to increase seed recruitment^55,98^. In addition, arthropods seem to have a positive relationship with rainfall seasonality as new flushing leaves increases the number of phytophagous insect species^96,99^. Nonetheless, we believe that the availability of different sorts of epiphytic substrates such as moss, bromeliads and others like leaves and flowers helps the arthropod community to be homogeneously distributed in the canopy and not to rely strictly on rainfall. Which in the end is beneficial to the woolly monkeys, as this allows them to forage constantly for this resource at any time in their home range.

Even though forest ripe fruit productivity can vary among years, in our study fruit productivity (389.62 kg/ha) was estimated to be very close to a recent previous study at the same location using the same method^57^ (392.28 kg/ha) and differed from previous estimates using visual phenological transects^16^ (248 kg/ha). Nevertheless, it must be noted that we were not able to sample during August and this might have lead to underestimation in fruit availability. One of the most productive species at the study site is most likely *Tapirira guianensis subandina*, which produces fruits mostly during this period of time^57^. Explaining why this fruit species was among the least selected, especially when *T. guianensis subandina* has been reported as one of the most important fruit sources in woolly monkeys’ diet^17,47^. Still, despite fruits being the most important item for this primate species diet, protein from arthropods is a very important component as it is fundamental for metabolic processes that might become limiting factors for growth, health, reproduction and survival^10^.

To our knowledge, this is the most complete list of identified arthropods in a woolly monkey species diet^23–25,27–29^ (Supplementary Data). With the help of metabarcoding, we have been able to confirm that woolly monkeys do eat spiders (Araneae), beetles (Coleoptera), fig wasps and ants (Hymenoptera), crickets (Orthoptera) and occasionally mantids (Mantodea), in addition we were able to considerably broaden the diet with the addition of moths (Lepidoptera), flies (Diptera), mites (Acariformes), scorpions (Scorpiones), cockroaches (Blattodea) and stick-bugs (Phasmatodea). Although, this technique does not allow us to assess which sequences within the diet might be a product of secondary predation (e.g. flies eaten by spiders, moths infested with flies, larvae infesting fruits)^100^. We were able to detect during the study infested fruits with some arthropods present in the woolly monkeys’ diet such as coleopteran larvae in ripe fruits of *Tapirira guianensis subandina* and *Saurauia brachybotrys*, and fig wasps inside figs of *Ficus americana* and *Ficus maxima*.

Unfortunately, there is no single sampling technique able to capture all types of arthropods without biases. For example, with our approach we were not able to collect many stick-bugs (Phasmatodea). Woolly monkeys seemed to select this insect the most in our study compared to its abundance, but perhaps this result is due to the lack of stick-bugs sampled in the canopy. Despite limitations, we still consider that combining the modified aerial composite traps^51^ made in this study with climbing trees to collect substrates, and using the fruit hanging traps was a better approach to sample the availability of resources compared to a previous study at Guácharos^16^. In contrast to Fonseca et al.^16^, this study shows similar patterns in the dietary composition of this monkey population and the constant arthropod availability over time. However, we noticed that by integrating different techniques and improving the sampling methods we could overcome the restrictions we had in that previous study. By having a higher resolution of the resource availability we were able to detect changes in the foraging patterns that previously we were only able to infer. For these reasons, we believe that this study explains, with a higher resolution. The variation of how fruit and arthropod availability influences the feeding ecology of the Colombian highland woolly monkey in their natural wild habitat.

In conclusion, one of our most important findings is that Colombian highland woolly monkeys do not select food stuffs randomly and opportunistically despite being generalist foragers. This primate species prefers and avoids some arthropod species relative to their availability. Additionally, we highlight other relevant findings such as broadening extensively the diet of this endemic Critically Endangered subspecies, and the positive relationship between arthropod feeding time with the availability of this item in the canopy. This raises questions about the actual nutritional gain of eating arthropods for such a large-bodied species. Further, Colombian highland woolly monkeys include a wide variety of arthropods in their diet, with Lepidopterans the most important over time, which makes it not only interesting in terms of the diet, but also in terms of the strategy they might have to degrade the hard exoskeletons of arthropods. Indeed, arthropods are an important food source for this population that not only broadens the diet, but also influences the behaviour of this population in periods of scarcity, where these monkeys invest more time foraging for this and other resources such as fall-back foods instead of resting more in order to save energy. Against our expectations, adults invested more time foraging for arthropods than juveniles, but not in the sort of arthropods they are able to capture and eat in the canopy, opening a debate whether woolly monkeys invest so much time foraging on arthropods because of their nutritional value, the monkeys social dynamics or a persistent strategy to reduce competition with other frugivorous species.

## Supplementary Information

Supplementary Table 1.

## Data Availability

The resulting DNA sequences were deposited in the European Nucleotide Archive (ENA) under the project accession number PRJEB51349.
