# Supplementary material for: Dietary preferences and feeding strategies of Colombian highland woolly monkeys
Source: Sci Rep. 2022 Aug 23;12:14364. doi: 10.1038/s41598-022-17655-5 (PMC9399098; doi:10.1038/s41598-022-17655-5)
Supplement: Supplementary file 1 — Supplementary Table 1. [file 41598_2022_17655_MOESM1_ESM.docx]

Supplementary Table 1. Invertebrate Amplicon Sequence Variants present in the scats of Colombian highland Woolly Monkeys (*Lagothrix lagotricha lugens*) at Cueva de los Guácharos National Park.

| Class | Order | Family | ASV Counts | Relative Abundance | % in Diet |
| --- | --- | --- | --- | --- | --- |
| Arachnida | Araneae | Anyphaenidae | 24 | 0.014 |  |
|  |  | Araneidae | 15 | 0.009 |  |
|  |  | Ctenidae | 1 | 0.001 |  |
|  |  | Linyphiidae | 4 | 0.002 |  |
|  |  | Oxyopidae | 2 | 0.001 |  |
|  |  | Pisauridae | 1 | 0.001 |  |
|  |  | Salticidae | 15 | 0.009 |  |
|  |  | Tetragnathidae | 4 | 0.002 |  |
|  |  | Theridiidae | 18 | 0.011 |  |
|  |  | Thomisidae | 5 | 0.003 |  |
|  |  | Others | 105 | 0.062 |  |
|  |  | **Total** | **194** | **0.114** | **11.37** |
| Insecta | Blattodea | Blattidae | 2 | 0.001 |  |
|  |  | Ectobiidae | 14 | 0.008 |  |
|  |  | Others | 13 | 0.008 |  |
|  |  | **Total** | **29** | **0.017** | **1.7** |
| Insecta | Coleoptera | Buprestidae | 1 | 0.001 |  |
|  |  | Carabidae | 5 | 0.003 |  |
|  |  | Cerambycidae | 4 | 0.002 |  |
|  |  | Chrysomelidae | 3 | 0.002 |  |
|  |  | Coccinelidae | 3 | 0.002 |  |
|  |  | Curculionidae | 31 | 0.018 |  |
|  |  | Dytiscidae | 3 | 0.002 |  |
|  |  | Elateridae | 2 | 0.001 |  |
|  |  | Scarabeidae | 1 | 0.001 |  |
|  |  | Staphylinidae | 4 | 0.002 |  |
|  |  | Zopheridae | 1 | 0.001 |  |
|  |  | Others | 60 | 0.035 |  |
|  |  | **Total** | **118** | **0.069** | **6.92** |
| Insecta | Diptera | Agromyzidae | 8 | 0.005 |  |
|  |  | Asilidae | 1 | 0.001 |  |
|  |  | Cecidomyiidae | 53 | 0.031 |  |
|  |  | Ceratopogonidae | 2 | 0.001 |  |
|  |  | Chironomidae | 15 | 0.009 |  |
|  |  | Culicidae | 3 | 0.002 |  |
|  |  | Drosophilidae | 4 | 0.002 |  |
|  |  | Muscidae | 6 | 0.004 |  |
|  |  | Mycetophilidae | 6 | 0.004 |  |
|  |  | Phoridae | 5 | 0.003 |  |
|  |  | Sarcophagidae | 3 | 0.002 |  |
|  |  | Sciaridae | 6 | 0.004 |  |
|  |  | Scyrphidae | 1 | 0.001 |  |
|  |  | Sepsidae | 3 | 0.002 |  |
|  |  | Simuliidae | 1 | 0.001 |  |
|  |  | Syrphidae | 7 | 0.004 |  |
|  |  | Tabanidae | 2 | 0.001 |  |
|  |  | Tachinidae | 24 | 0.014 |  |
|  |  | Tipulidae | 2 | 0.001 |  |
|  |  | Others | 127 | 0.074 |  |
|  |  | **Total** | **279** | **0.164** | **16.35** |
| Insecta | Hemiptera | Aphididae | 8 | 0.005 |  |
|  |  | Cicadellidae | 8 | 0.005 |  |
|  |  | Cicadidae | 4 | 0.002 |  |
|  |  | Clastopteridae | 3 | 0.002 |  |
|  |  | Coreidae | 1 | 0.001 |  |
|  |  | Geocoridae | 2 | 0.001 |  |
|  |  | Membracidae | 24 | 0.014 |  |
|  |  | Miridae | 4 | 0.002 |  |
|  |  | Notonectidae | 4 | 0.002 |  |
|  |  | Pentatomidae | 11 | 0.006 |  |
|  |  | Others | 59 | 0.035 |  |
|  |  | **Total** | **128** | **0.075** | **7.5** |
| Insecta | Hymenoptera | Agaonidae | 1 | 0.001 |  |
|  |  | Aphelinidae | 3 | 0.002 |  |
|  |  | Braconidae | 14 | 0.008 |  |
|  |  | Colletidae | 2 | 0.001 |  |
|  |  | Crabronidae | 2 | 0.001 |  |
|  |  | Eulophidae | 8 | 0.005 |  |
|  |  | Eurytomidae | 7 | 0.004 |  |
|  |  | Formicidae | 2 | 0.001 |  |
|  |  | Ichneumonidae | 7 | 0.004 |  |
|  |  | Platygastridae | 1 | 0.001 |  |
|  |  | Pteromalidae | 15 | 0.009 |  |
|  |  | Vespidae | 2 | 0.001 |  |
|  |  | Others | 34 | 0.020 |  |
|  |  | **Total** | **98** | **0.057** | **5.74** |
| Insecta | Lepidoptera | Bombycidae | 8 | 0.005 |  |
|  |  | Cosmopterigidae | 3 | 0.002 |  |
|  |  | Crambiidae | 16 | 0.009 |  |
|  |  | Dalceridae | 2 | 0.001 |  |
|  |  | Depressaridae | 5 | 0.003 |  |
|  |  | Erebidae | 37 | 0.022 |  |
|  |  | Gelechiidae | 9 | 0.005 |  |
|  |  | Geometridae | 151 | 0.089 |  |
|  |  | Hesperiidae | 19 | 0.011 |  |
|  |  | Lycaenidae | 5 | 0.003 |  |
|  |  | Noctuidae | 37 | 0.022 |  |
|  |  | Notodontidae | 18 | 0.011 |  |
|  |  | Nymphalidae | 49 | 0.029 |  |
|  |  | Oecophoridae | 7 | 0.004 |  |
|  |  | Papilionidae | 6 | 0.004 |  |
|  |  | Pyralidae | 27 | 0.016 |  |
|  |  | Riodinidae | 4 | 0.002 |  |
|  |  | Saturniidae | 20 | 0.012 |  |
|  |  | Sphingidae | 20 | 0.012 |  |
|  |  | Thyrididae | 3 | 0.002 |  |
|  |  | Tortricidae | 19 | 0.011 |  |
|  |  | Uraniidae | 1 | 0.001 |  |
|  |  | Urodidae | 7 | 0.004 |  |
|  |  | Others | 212 | 0.124 |  |
|  |  | **Total** | **685** | **0.402** | **40.15** |
| Insecta | Orthoptera | Acrididae | 4 | 0.002 |  |
|  |  | Gryllidae | 14 | 0.008 |  |
|  |  | Tettigonidae | 9 | 0.005 |  |
|  |  | Trigonidiidae | 1 | 0.001 |  |
|  |  | Others | 18 | 0.011 |  |
|  |  | **Total** | **46** | **0.027** | **2.7** |
| Insecta | Phasmatodea | Diapheromeridae | 4 | 0.002 |  |
|  |  | Phasmatidae | 8 | 0.005 |  |
|  |  | Pseudophasmatidae | 2 | 0.001 |  |
|  |  | Others | 3 | 0.002 |  |
|  |  | **Total** | **17** | **0.010** | **1.00** |
| Insecta | Ephemeroptera | - | 3 | 0.002 | **0.18** |
| Insecta | Mantodea | - | 1 | 0.001 | **0.06** |
| Insecta | Neuroptera | - | 5 | 0.003 | **0.29** |
| Arachnida | Sarcoptiformes | - | 7 | 0.004 | **0.41** |
| Arachnida | Scorpiones | Euscorpiidae | 1 | 0.001 | **0.06** |
| Insecta | Tichoptera | - | 4 | 0.002 | **0.23** |
| Arachnida | Trombidiformes | - | 16 | 0.009 | **0.94** |
|  | Others | No ID | 75 | 0.044 | **4.40** |
|  |  |  |  |  |  |
